# An unusual presentation of intestinal duplication mimicking torsion of Meckel’s diverticulum: a rare report of a pediatric case

**DOI:** 10.1186/s40792-022-01409-6

**Published:** 2022-03-28

**Authors:** Makoto Matsukubo, Mitsuru Muto, Chihiro Kedoin, Mayu Matsui, Masakazu Murakami, Koshiro Sugita, Keisuke Yano, Shun Onishi, Toshio Harumatsu, Koji Yamada, Waka Yamada, Tatsuru Kaji, Satoshi Ieiri

**Affiliations:** 1 Department of Pediatric Surgery, Research Field in Medical and Health Sciences, Medical and Dental Area, Research and Education Assembly, Kagoshima University, 8-35-1, Sakuragaoka, Kagoshima 8908520 Japan; 2 Clinical Training Center, Kagoshima University Hospital, Kagoshima, Japan; 3 Department of Pediatric Surgery, Kurume University School of Medicine, Kurume, Japan

**Keywords:** Enteric duplication, Intestinal duplication, Torsion, Meckel’s diverticulum, Acute abdomen

## Abstract

**Background:**

Enteric duplication is a congenital disease that occurs throughout the entire gastrointestinal tract. Although it may sometimes cause intestinal volvulus, a few reports have described cases of enteric duplication twisted on itself. We experienced a rare pediatric case of long-segment tubular ileal duplication showing torsion. Torsion of enteric duplication is extremely rare. We herein report a pediatric case showing unusual torsion of ileal duplication requiring emergency surgery.

**Case presentation:**

A 3-year-old boy presented with abdominal pain and vomiting. Contrast-enhanced computed tomography (CT) revealed a cystic luminal structure with a blind end and fluid collection in the pelvic cavity. CT also showed no findings of ileus or intestinal dilatation except for a cystic luminal structure. The preoperative diagnosis was torsion of Meckel’s diverticulum. The patient underwent emergent explorative diagnostic laparoscopy. As a result, a necrotic luminal structure and bloody ascites were recognized, and small-scale laparotomy was performed. Long-segment ileal duplication was recognized. The long-segment tubular ileal duplication shared the anti-mesenteric side of the intestinal wall along one-third of its length. The residual two-thirds of its length was free from the ileum and its blind end was twisted in a manner that looked similar to Meckel’s diverticulum. Normal ileum and the duplication, including the twisted necrotic portion, were resected, and ileal anastomosis was performed. The postoperative course was uneventful. A pathological examination confirmed the definitive diagnosis of enteric duplication.

**Conclusions:**

We reported the unusual presentation of intestinal duplication mimicking torsion of Meckel’s diverticulum. Enteric duplication shows various clinical symptoms and presentations. We must understand that the classification of digestive enteric duplication is diverse with a variety of associated clinical symptoms.

## Background

Enteric duplication is a congenital disease that occurs throughout the entire gastrointestinal tract. Although it may cause intestinal volvulus, there are few reports of enteric duplication itself being twisted.

We herein report a pediatric case in which the diverticulum-like site of tubular intestinal duplication was twisted and necrotic. Torsion of enteric duplication is extremely rare, and this case is reported along with a review of the literature.

## Case presentation

**Fig. 1:**
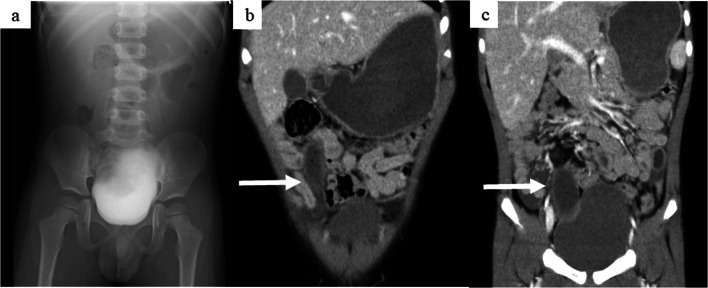
Abdominal X-ray and contrast-enhanced CT. **a** An abdominal X-ray did not show dilation of intestine and findings of ileus. **b**, **c** From the right middle abdomen to the bottom of the pelvis, we detected a gastrointestinal-like lesion (arrow) that was blind with fluid retention inside

A 3-year-old boy presented with complaints of acute abdominal pain. The patient had visited the primary care pediatric clinic the day after the onset and been diagnosed with viral gastroenteritis and received medication conservatively. However, the abdominal pain persisted, so the patient was referred by his primary care physician to the previous local hospital. An abdominal X-ray did not show dilation of intestine and findings of ileus (Fig. 1a). The patient underwent contrast-enhanced computed tomography (CT), which revealed a cystic lesion similar to the structure of the gastrointestinal tract and ascites retention in the abdominal cavity (Fig. 1b, c). The patient was therefore transferred to our hospital for acute abdomen. He had a fever and showed abdominal distension and tenderness with peritoneal signs in the right lower abdomen. Blood sampling showed an elevated white blood cell count and CRP (Table 1).

**Table 1:** Blood test on admission

White blood cell, uL	20,800
Hemoglobin, g/dl	12.0
Platelet, uL	388,000
C-reactive protein, mg/dl	5.79
Aspartate aminotransferase, U/L	29
Alanine transaminase, U/L	11
γ-Guanosine triphosphate, U/L	9
P-Amylase, U/L	40
Total-bilirubin, mg/dl	0.8
Albumin, g/dl	4.3
Blood urea nitrogen, mg/dl	8.0
Creatinine, mg/dl	0.21
Creatinine kinase, U/L	100
Lactic acid, mmol/l	0.8
Glucose, mg/dl	125
Prothrombin time-international normalized ratio	1.21
Activated partial thromboplastin time, sec	37.8

We reviewed the contrast-enhanced CT findings again. There were no findings of ileus or intestinal dilatation except for the cystic luminal structure. The blood supply of the entire gastrointestinal tract was also preserved, except for the cystic luminal structure. Based on the clinical symptoms and these imaging findings, the preoperative diagnosis was torsion of Meckel’s diverticulum.

**Fig. 2:**
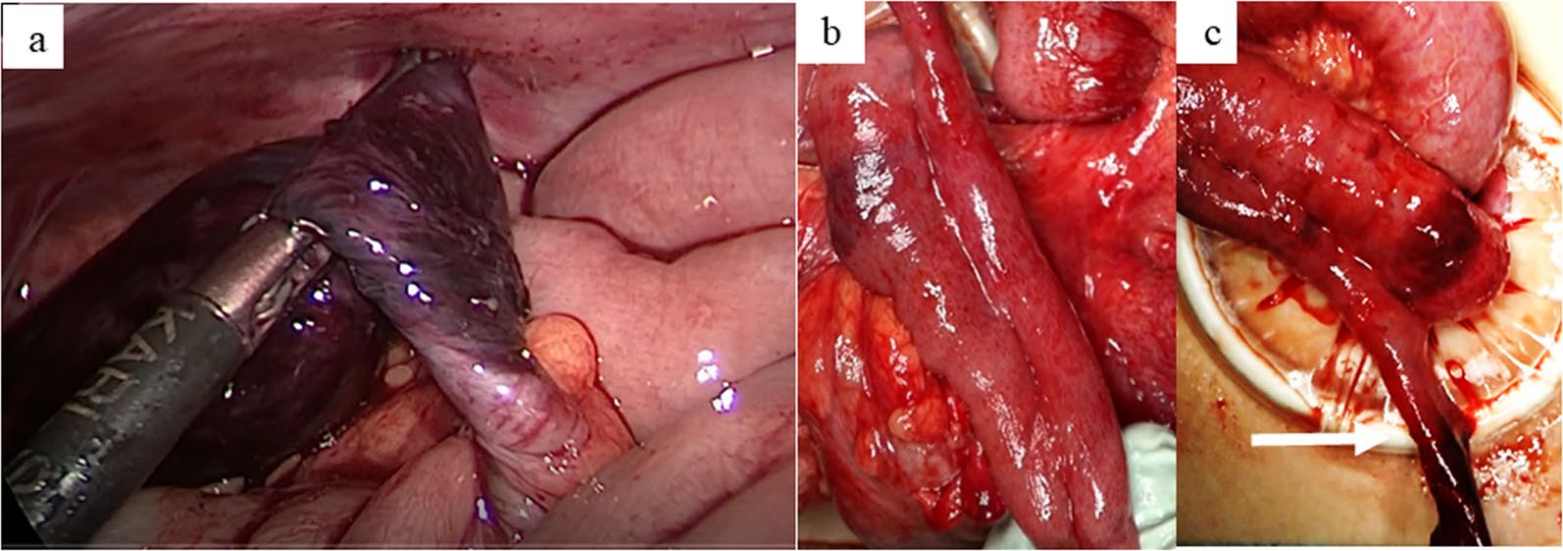
Intraoperative findings. **a** A strangulated necrotic tubular structure was recognized to demonstrate torsion. Its root was continuous with the intestinal tract and was twisted. **b**, **c** Intestinal duplication was found along the normal intestinal tract. It shared a wall with the small intestine and ran in parallel for 15 cm, but its anal side showed a diverticulum-like form (arrow) and was twisted

We decided to perform exploratory laparoscopic inspection to obtain a definitive diagnosis. Exploratory laparoscopy revealed that the dark-colored twisted cystic luminal structure was located at the right lower abdomen, and its root seemed to be continuous with the small intestine (Fig. 2a). After releasing the 720° clockwise twisting, the cystic luminal structure was confirmed to have branched off of the small intestine. However, the connected formation was not that of Meckel’s diverticulum, but rather duplication.

Small-scale laparotomy using the umbilical trocar wound was then performed to clarify the pathophysiological mechanism. The entire small intestine was extracted through the wound retractor. A long segment of tubular ileal duplication was recognized at 120 cm from the oral side of the ileocecal region (Fig. 2b). The long-segment tubular ileal duplication shared the anti-mesenteric side of the intestinal wall along one-third of its length. The residual two-thirds of its length was free from the ileum and twisted in a manner similar to Meckel’s diverticulum. Thin pedicle-like structure was recognized at the root of duplication cyst. Blood supply was thought to be kept by both thin pedicle and intramural blood flow. The diverticulum-like part was necrotic due to the twisting (Fig. 2c). The intraoperative diagnosis was torsion of intestinal duplication. The duplicated portion was resected along with the normal ileum, and intestinal anastomosis was performed with one layer. An abdominal drain was not placed, and the wound was closed in layers.

**Fig. 3:**
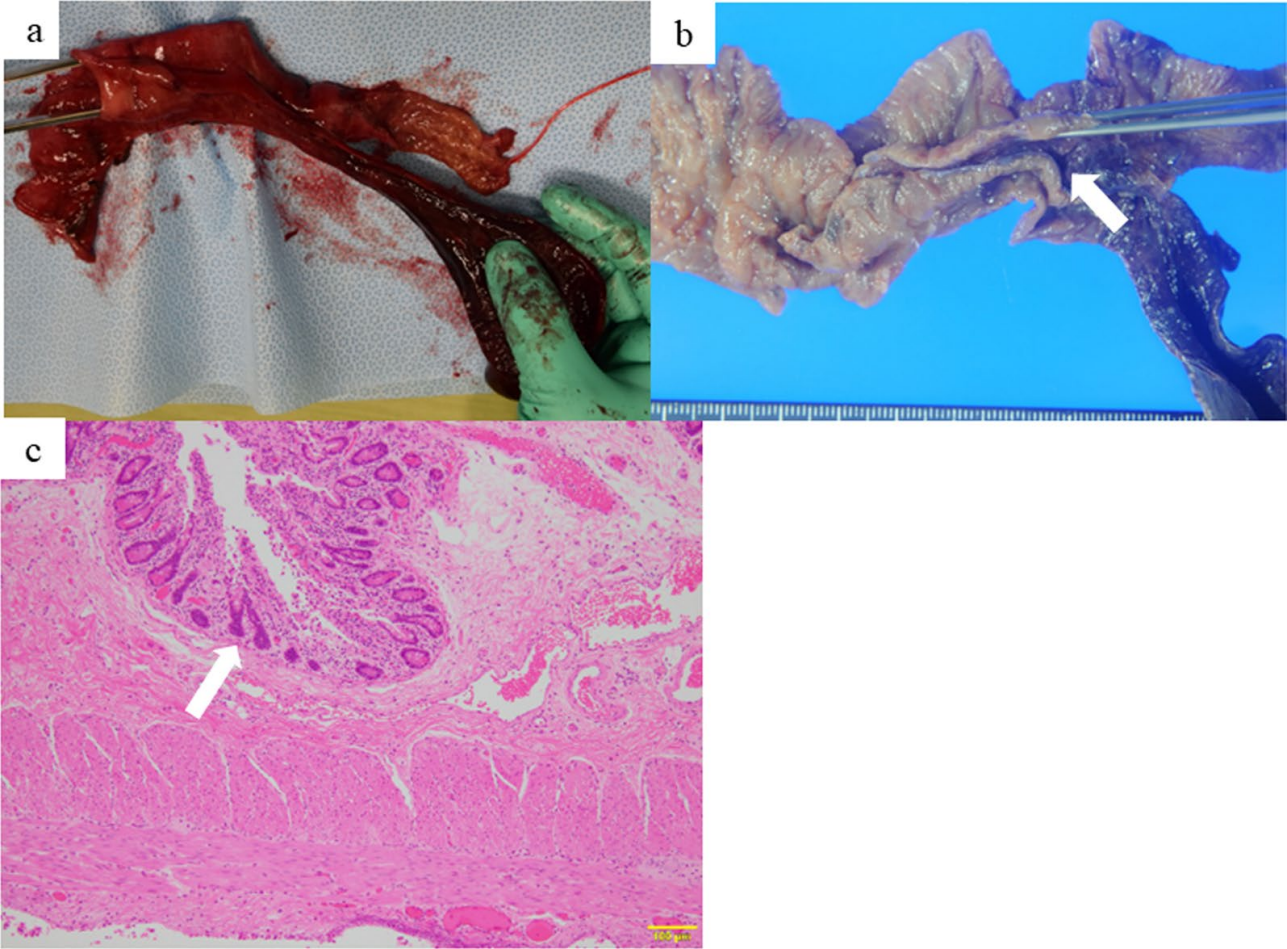
Excised specimen and pathological findings.** a**, **b** The overall picture of the intestinal duplication, partially protruding like a diverticulum and showing necrosis due to twisting. **b** The inner surface of the intestinal duplication was covered with intestinal mucosa. (arrow). **c** Hematoxylin–eosin staining of resected specimen. The inner surface of the intestinal duplication was covered with intestinal epithelium (arrow)

The excised specimen is shown in Fig. 3a. The pathological findings revealed that the luminal structure shared a muscular layer with the normal ileum and had an inner surface covered with intestinal epithelium, which was compatible with intestinal duplication, as shown in Fig. 3b and c.

The postoperative course was uneventful, and the patient left our hospital on postoperative day 14. He showed no adverse events in 3 years of follow-up.

## Discussion

The term enteric duplication was first used by Fitz [1], but was not widely used until it was popularized by Ladd in the 1930s [2], with further classifications by Gross in the 1950s [3]. The reported incidence is 1:4.500 births, and most duplications are recognized in children (antenatally or within first 2 years of life), with fewer than 30% of all duplications diagnosed in adults. Approximately 75% of duplications have been reported to be located within the abdominal cavity, whereas the remaining cases are intrathoracic (20%) or thoracoabdominal (5%). Although the exact etiology is unknown, several theories have been proposed, including a split notochord (currently the most favored), partial twinning, persistent embryological diverticula and aberrant luminal re-canalization. Other rare causes include intrauterine trauma and hypoxia [4, 5].

Many authors have reported variable clinical presentations depending on the location, size and other factors, such as the presence of ectopic mucosa, communication with adjacent bowel or inflammation. Infants and neonates sometimes show abdominal pain, nausea, vomiting, bleeding, abdominal distension, abdominal mass, obstruction and intussusception. Some duplication cysts may remain asymptomatic until adulthood [6].

Duplication can sometimes cause intestinal volvulus, but there have been a few reports in which the enteric duplication itself became twisted. A search on PubMed for the period 1950–2021 using "enteric duplication", "volvulus" and "torsion" as search words revealed four cases of volvulus of the enteric duplication itself. Table 2 shows the five total cases of volvulus of the enteric volvulus itself, including our present case [7–10]. The patient age ranged from 1 to 53 years old. The main complaint was abdominal pain in all cases, and biliary vomiting was also observed in cases 1 and 2. Imaging revealed dilation of the small intestine and cystic structures. The size varied from 10 to 70 cm. In case 1, the duplicated colonic segment demonstrated torsion over the partially duplicated appendix. Cases 2 and 3 involved a completely isolated duplication cyst in the ileal mesentery, and Case 4 involved tubular intestinal duplication contralateral to the mesentery, similar to our case.

**Table 2:** Reported cases of twisted enteric duplication

Case	Author	Year	Age (years)	Sex	Symptom	Imaging examination findings	Size (cm)	Operative findings	Surgical procedure
1	Oğuzkurt et al [7]	2004	2	Male	Abdominal pain Distension Bilious vomiting	A tubular cystic structure	10 × 3 × 3	A colonic duplication associated with duplicated appendix	Simple appendectomy
2	Pant et al. [8]	2012	1	Male	Abdominal pain Distension Bilious vomiting	Small bowel volvulus with gangrene	25 × 9	A completely isolated duplication cyst in the ileal mesentery	Excision of the enteric duplication cyst along with the adjacent ileum
3	Xiao-Ming et al. [9]	2018	20	Male	Abdominal pain Distension Vomiting	Multiple evidently dilated cystic small-bowel loops	70 × 8	A completely isolated duplication cyst in the ileal mesentery	Excision of the enteric duplication cyst
4	Huang et al [10]	2018	53	Male	Abdominal pain Distension	A cystic intestinal dilation	Unknown	An intestinal duplication	Excision of the intestinal duplication cyst along with the adjacent ileum
5	Ou case	2021	3	Male	Abdominal pain Vomiting	A tubular cystic structure	30	A tubular duplication with some diverticulum structures	Excision of the intestinal duplication cyst along with the adjacent ileum

A search on PubMed for the period 1950–2021 using "enteric duplication", "volvulus", and "torsion" as search words revealed four cases of volvulus of the enteric duplication itself

In general, intestinal duplication is located on the mesenteric side and shares a smooth muscle wall with the adjacent gastrointestinal tract. Because the enteric duplication is fixed between the mesentery and the adjacent intestinal tract, it seems difficult to twist. However, in Case 1, the colon duplication was attached to the periphery of the appendix duplication. In addition, the duplication was only in contact with the mesentery with a thin stalk-like structure in Cases 2 and 3. In Cases 4 and 5, diverticulum-like intestinal duplication protruded from the opposite side of the mesentery.

The twisting is considered to be due to the enteric duplication having a structure with good mobility. Ladd et al. proposed a concept of three characteristics: (1) a well-developed smooth muscle coat; (2) mucosal lining found within some portion of the alimentary tract; and (3) contiguity to any segment of the alimentary tract [2]. It is also said that the enteric duplication is usually located on the mesenteric side. However, in recent years, there have been reports of varied cases, such as those that are isolated and those that occur on the opposite side of the mesentery.

The broadly accepted classification of intestinal duplication consists of five types: intestinal membrane type, intestinal wall cyst type, extra-intestinal cyst type, extra-intestinal tubular type and solitary type [11]. The prevalence of gastrointestinal duplication varies, but it is more common in the ileum and ileocolic segment than in other sites. Intestinal duplication is usually cystic or tubular in nature and often continuous with the regular bowel wall, and it shares the muscle and mucosal layer [12]. Li et al. classified enteric duplication into two types according to the pattern of the vascular supply in relation to the duplication and involved bowel [13]. The authors noted that duplications of small intestine have a relatively independent vascular supply and can be resected without the adjacent bowel. Our case seems to be a pattern (Type1c) in which the duplication is located on one side of the mesentery as described by long li et al. Blood perfusion of the conjoined segment was supplied from thin pedicle of the mesentery and those of the residual two-thirds segment was supplied from intramural blood flow. Hung et al. also suggested broad variations in the current anatomical classification [10]. Understanding the different potential forms of enteric duplication can help diagnose enteric duplication based on clinical symptoms and imaging findings.

Our case shared characteristics with the long-segment tubular type and was located at the anti-mesenteric side, and two-thirds of it was also free from the adjacent bowel. The free side had a blind end and the complete structure of the intestinal wall, so the duplication might have had some degree of motility. As a result, torsion would have been able to be induced that mimicked torsion of Meckel’s diverticulum around the root on the free side without the involvement of the mesentery.

## Conclusions

We experienced a rare case in which a portion of intestinal duplication located on the anti-mesenteric side protruded with a diverticulum shape and had become twisted. We should consider enteric duplication when encountering cases of cystic lesions presenting with twisting mimicking torsion of Meckel’s diverticulum. Enteric duplication can present with a variety of clinical symptoms.

## Data Availability

The data that support the findings of this study are available from the corresponding author upon reasonable request.

## References

[1] Fitz RH (1884). Persistent omphalomesenteric remains: their importance in the causation of intestinal duplication, cyst formation and obstruction. Am J Med Sci.

[2] Ladd WE, Gross RE (1940). Surgical treatment of duplications of the alimentary tract enterogenous cysts, enteric cysts or ileum duplex. Surg Gynecol Obstet.

[3] Gross RE. Duplications of the alimentary tract. In: The Surgery of Infancy and Childhood. 1953;221–245.

[4] Schalamon J, Schleef J, Hollwarth ME (2000). Experience with gastro-intestinal duplications in childhood. Langenbecks Arch Surg.

[5] Merrot T, Anastasescu R, Pankevych T, Tercier S, Garcia S, Alessandrini P (2006). Duodenal duplications. Clinical characteristics, embryological hypotheses, histological findings, treatment. Eur J Pediatr Surg.

[6] Macpherson RI (1993). Gastrointestinal tract duplications: clinical, pathologic, etiologic, and radiologic considerations. Radiographics.

[7] Sharma S, Yadav AK, Mandal AK, Zaheer S, Yadav DK, Samie A (2015). Enteric duplication cysts in children: a clinicopathological dilemma. J Clin Diagn Res.

[8] Oğuzkurt P, Oğuzkurt L, Kayaselcuk F, Oz S (2004). An unusual cause of acute abdomen: torsion of colonic duplication over a duplicated appendix. Pediatr Surg Int.

[9] Xiao-Ming A, Jin-Jing L, Li-Chen H, Lu-Lu H, Xiong Y, Hong-Hai Z (2018). A huge completely isolated duplication cyst complicated by torsion and lined by 3 different mucosal epithelial components in an adult: a case report. Medicine (Baltimore).

[10] Huang ZH, Wan ZH, Vikash V, Vikash S, Jiang CQ (2018). Report of a rare case and review of adult intestinal duplication at the opposite side of mesenteric margin. Sao Paulo Med J.

[11] Fuyou H, Hong Y, Yan S. The diagnosis and classification of intestinal duplication (in Chinese). Chinese J. Pediatr. Surg. 1997; 18: 91–93. Available from: http://eng.med.wanfangdata.com.cn/PaperDetail.aspx?qkid=xrwk&qcode=xrwk199702010#.

[12] Sinha A, Ojha S, Sarin YK (2006). Completely isolated, noncontiguous duplication cyst. Eur J Pediatr Surg.

[13] Li L, Zhang JZ, Wang YX (1998). Vascular classification for small intestinal duplications: experience with 80 cases. J Pediatr Surg.

